# Posture influences patient cough rate, sedative requirement and comfort during bronchoscopy: An observational cohort study

**DOI:** 10.1186/1745-9974-7-9

**Published:** 2011-11-10

**Authors:** Ivan T Ling, Francesco Piccolo, Siobhain A Mulrennan, Martin J Phillips

**Affiliations:** 1 Department of Respiratory Medicine, Sir Charles Gairdner Hospital, Perth, Western Australia, Australia

**Keywords:** bronchoscopy, posture, cough, sedation, hypoxia

## Abstract

**Objectives:**

To investigate differences between semi-recumbent and supine postures in terms of cough rate, oxygen desaturation, sedative use, and patient comfort during the initial phase of bronchoscopy.

**Methods:**

Consecutive bronchoscopy patients (n = 69) participated in this observational cohort study. Posture was determined by the bronchoscopist's usual practice. Patient demographics, spirometry, pulse, and SpO_2 _were recorded. The initial phase was defined as the time from bronchoscopy insertion to visualisation of both distal main bronchi. Cough rate, peak pulse, nadir SpO_2_, oxygen supplementation, and sedative use during the initial phase were recorded. A post-procedure questionnaire was administered to the patient and the attending nurse.

**Results:**

36 patients had bronchoscopy in the semi-recumbent posture, 33 in the supine posture. 3 of 5 bronchoscopists performed in both postures. There were no differences in baseline parameters between the groups. The semi-recumbent posture resulted in significantly less cough (mean (SD) 3.6 (2.3) vs. 6.1 (4.5) coughs/min, p = 0.007) and less fentanyl use (70 (29) vs. 88 (28) mcg, p = 0.011) in the initial phase. There were no significant differences in the nadir SpO_2_, fall in SpO_2_, oxygen supplementation, or increase in pulse rate between the groups. On 100 mm visual analogue scale, nurse perception of patient discomfort was lower in the semi-recumbent position (23 (21) vs. 39 (28) mm, p = 0.01), and there was a trend towards less patient perceived cough in the semi-recumbent group (28 (25) vs. 40 (28) mm, p = 0.06).

**Conclusions:**

Bronchoscopy performed in the semi-recumbent posture results in less cough and sedative requirement, and may improve patient comfort.

## Background

Diagnostic flexible bronchoscopy is a common procedure performed for a wide variety of indications. It has a low rate of mortality and major complications, estimated at around 0.01-0.04% and a 0.08-0.3% respectively [1-4]. Major complications related to the procedure include respiratory depression, pneumonia, pneumothorax, arrhythmias, and pulmonary oedema [2,4-6]. Minor complications can include vasovagal reactions, bronchospasm, fever, haemorrhage, airway obstruction, nausea, and vomiting [5].

Hypoxemia is known to occur during flexible bronchoscopy [6-8]. It is likely that a number of mechanisms contribute to this, including ventilation-perfusion mismatch and hypoventilation secondary to sedative use [9]. In addition, there may be a relationship between hypoxemia and patient posture during bronchoscopy. Bronchoscopy is typically performed either in the supine or the semi-recumbent (or sitting) posture, and the choice of posture is largely a matter of preference or habit [10]. A number of studies have investigated the influence of posture on the degree of hypoxemia during flexible bronchoscopy, although outcomes have been mixed [10-12]. One particular report found that the degree of oxygen desaturation during bronchoscopy correlated with baseline oxygen saturation (SpO_2_) and percent predicted FEV1 [12].

In this study, we aimed to examine differences between the supine and semi-recumbent postures during bronchoscopy in terms of degree of oxygen desaturation and oxygen supplementation required, cough rate, increase in pulse rate, and amount of sedative medication required during the initial phase of the procedure. We also aimed to examine differences between postures in terms patient and nurse perceived discomfort using a questionnaire, and the influence of measures of ventilatory capacity and baseline SpO_2 _on procedural oxygen desaturation.

## Methods

A prospective observational cohort study was performed at the diagnostic bronchoscopy unit at Sir Charles Gairdner Hospital between June and September 2010. Consecutive patients planned for diagnostic bronchoscopy were recruited for the study after providing informed consent. The study was approved by the local human research ethics committee.

Demographic and clinical information collected included age, gender, smoking status, admission status, procedure indication, baseline SpO_2_, percent predicted FEV1 (%FEV1), percent predicted FVC (%FVC), and bronchoscopy operator, posture and route. The choice of posture was determined by the operator's usual practice. The supine posture was defined as bed-head elevation angle of 0-15 degrees, whilst the semi-recumbent posture was defined as an angle of 45-60 degrees. Patients were excluded if they required supplemental oxygen prior to the procedure.

### Pre-procedural protocol

Prior to the commencement of the procedure, patients gargled 10 mL of 2% lignocaine followed by two sprays of CoPhenylcaine Forte (containing lignocaine 50 mg/mL and phenylephrine 5 mg/mL; ENT Technologies, East Malvern, VIC, Australia) to the pharynx, and 2 mL of 2% lignocaine gel into each nostril for those who are elected for nasal intubation. Patients were commenced on 2 L/min of supplemental oxygen via nasal prongs, and the flow was titrated up at 2 L/min increments during the procedure if SpO2 fell below 90%. Prior to the introduction of the bronchoscope, sedation with 1-3 mg of midazolam and 25-50 mcg of fentanyl was given intravenously. Amounts of sedative medication were titrated at the discretion of the operator to achieve optimal sedation and anti-tussive effect, with further doses given during bronchoscopy as required. Suggested initial doses at our institution are 3 mg and 50 mcg of midazolam and fentanyl respectively for patients aged below 70 years, 2 mg and 50 mcg for patients 70-80 years, and 1 mg and 25 mcg for patients older than 80 years.

### Bronchoscopy protocol

Following visualisation of the vocal cords, two aliquots of 2 mL of 2% lignocaine were applied, and the operator waited for 30 seconds prior to attempting tracheal intubation. Three further 2 mL aliquots of 2% lignocaine were then applied at the distal trachea, and into both main bronchi, for a total dose of 200 mg of lignocaine. The initial phase of the procedure was defined as the time from the introduction of the bronchoscope into the patient's mouth or nostril to the visualization of the distal ends of the main bronchi. During the procedure, a separate investigator performed a recording of the patient's cough on a sound recorder (Digital Pocket Memo 9600, Philips Australia, North Ryde, NSW), and recorded the amount of sedative medication used, nadir SpO_2_, peak pulse rate, and oxygen supplementation given up to the end of the initial phase of the procedure.

### Post-procedural protocol

**Figure 1:**
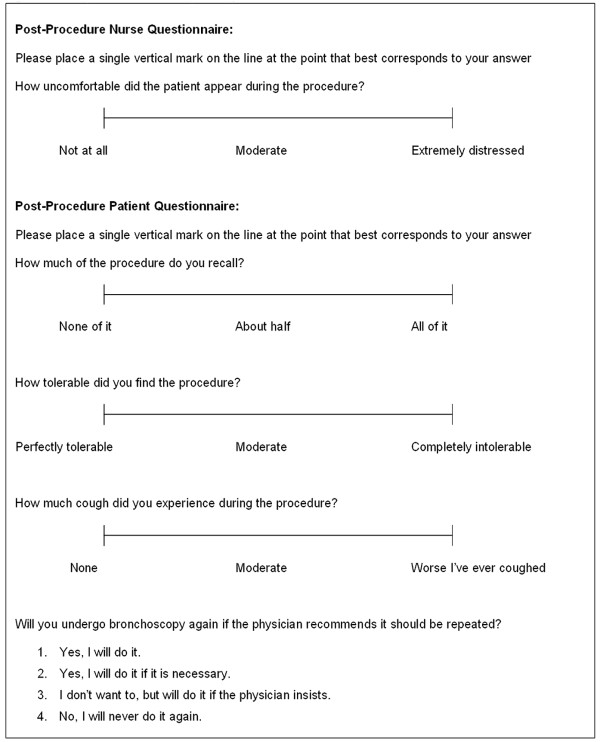
**Post-procedural nurse and patient questionnaire**.

After the procedure, attendant nursing staff provided an estimate of perceived patient discomfort on a 100 mm visual-analogue scale (VAS) (Figure 1). When the patient had recovered from sedation (usually after 60 minutes post-procedure), a patient questionnaire was administered (Figure 1). Two study investigators reviewed the sound recording to count the number of coughs heard. Coughs were defined as a forced expiratory manoeuvre, usually against a closed glottis and associated with a characteristic sound, as recommended by European Respiratory Society and British Thoracic Society guidelines [13,14]. Discordant results were reviewed and a consensus arrived at.

### Analysis

Results are expressed as mean ± SD or number (percentage) as stated. Paired t-tests or the Fisher exact test were used for comparisons between groups as appropriate. Pearson correlation was used to assess the relationships of %FEV1, %FVC, and baseline SpO_2 _with procedural nadir SpO_2 _and fall in SpO_2_.

## Results

69 patients participated in the study, with 33 patients undergoing bronchoscopy in the supine posture and 36 patients in the semi-recumbent posture. Demographics, clinical data, and intra- and post-procedural observations for the total population and for groups divided by posture are summarised in Table 1. There was no statistically significant difference between the supine and semi-recumbent groups in baseline demographic and clinical data, including age, gender, smoking status, resting pulse and SpO_2_, %FEV1, and %FVC.

**Table 1:** Demographics, clinical data, and procedural observations for the study population and for groups by procedural posture.

	All patients	Supine	Semi-recumbent	p-value*
**n**	**69**	**33**	**36**	

Age	62 ± 14	64 ± 16	61 ± 13	NS
Males (%)	42 (61)	19 (58)	23 (64)	NS
Smoking status				
- Current smokers (%)	15 (22)	9 (27)	6 (17)	NS
- Ex-smokers (%)	33 (48)	16 (49)	17 (47)	NS
- Non-smokers (%)	21 (30)	8 (24)	13 (36)	NS
Inpatients (%)	26 (38)	11 (33)	15 (42)	NS
Baseline SpO_2_	97.0 ± 2.2	96.9 ± 2.4	97.1 ± 1.9	NS
Baseline pulse rate (/min)	77 ± 13	76 ± 13	77 ± 13	NS
% predicted FEV1	70 ± 19	67 ± 21	74 ± 17	NS
% predicted FVC	78 ± 17	79 ± 19	77 ± 14	NS
Oral route (%)	31 (45)	15 (45)	17 (47)	NS
				
Initial phase (min)	3.7 ± 1.3	3.9 ± 1.5	3.6 ± 1.1	NS
Cough rate (/min)	4.8 ± 3.7	6.1 ± 4.5	3.6 ± 2.3	0.007
Sedative use				
- Midazolam (mg)	3.5 ± 1.7	4.0 ± 2.0	3.1 ± 1.2	0.029
- Fentanyl (mcg)	79 ± 30	88 ± 28	70 ± 29	0.011
Nadir SpO_2_	93.4 ± 4.6	93.3 ± 4.3	93.6 ± 4.9	NS
Fall in SpO_2_	3.6 ± 4.2	3.6 ± 3.6	3.6 ± 4.7	NS
Peak pulse rate (/min)	92 ± 16	91 ± 17	94 ± 15	NS
Rise in pulse rate (/min)	16 ± 13	16 ± 12	16 ± 14	NS
Maximal O_2 _use (L/min)	3.0 ± 1.8	3.0 ± 2.1	3.0 ± 1.5	NS
				
Questionnaire^†^				
- Nurse VAS discomfort	31 ± 26	39 ± 28	23 ± 21	0.01
- Patient VAS recall	49 ± 35	51 ± 35	47 ± 36	NS
- Patient VAS discomfort	24 ± 23	22 ± 23	27 ± 23	NS
- Patient VAS cough	33 ± 27	40 ± 28	28 ± 25	0.067
- Patient 1-4 repeat	1.2 ± 0.4	1.2 ± 0.4	1.1 ± 0.4	NS

Values shown as mean ± SD or number (%).

* p-values shown for comparisons between supine and semi-recumbent groups.

^† ^VAS responses measured in mm from the left end of the visual analogue scale.

FEV1, forced expiratory volume in 1 second; FVC, forced vital capacity; SpO_2_, oxygen saturation; VAS, visual analogue scale; NS, not significant at > 0.05.

During the initial phase of the procedure the semi-recumbent group had a lower cough rate and use of sedative medications compared with the supine group (Table 1). There was no statistically significant difference in the length of the initial phase, nadir SpO_2_, fall in SpO_2_, peak pulse rate, rise in pulse rate, and maximum oxygen supplementation given between the groups.

**Figure 2:**
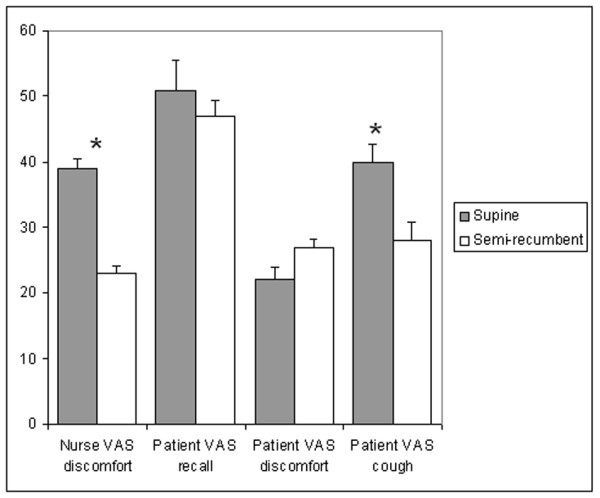
**Post-procedure visual analogue scale (VAS) questionnaire results for supine and semi-recumbent postures**. * indicates statistically significant difference.

Post-procedure VAS questionnaire results for the two postures are presented in Table 1 and Figure 2. On the post-procedure questionnaire, there was lower level of nurse-perceived patient discomfort and a trend towards less patient-perceived cough (p = 0.067) on VAS in the semi-recumbent group. There was no significant difference between groups in the level of patient recall of the procedure, perceived discomfort, and willingness to undergo a repeat procedure.

Three of five operators performed procedures in both the supine and semi-recumbent postures. Although numbers for each individual operator were too few for meaningful statistical comparison, results for these operators demonstrated lower mean cough rate (mean (SD) of 6.1 (4.5) vs. 3.8 (1.8) coughs/min in supine vs. semi-recumbent, p = 0.01) and fentanyl use in the semi-recumbent posture (88 (28) vs. 65 (39) mcg, p = 0.03), and a trend toward lower midazolam use (4.0 (2.0) vs. 3.3 (1.3) mg, p = 0.13).

**Figure 3:**
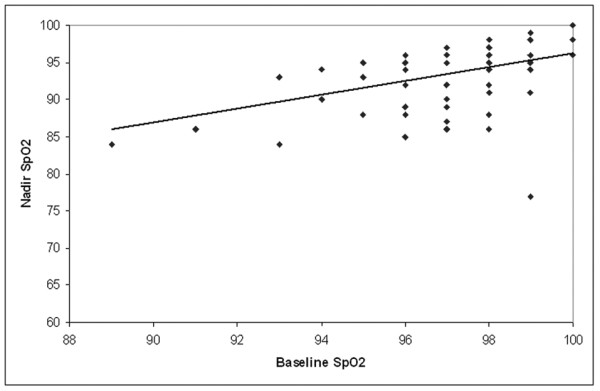
**Procedural nadir oxygen saturation (SpO_2_) versus baseline SpO_2 _in study subjects**. Line of best fit shown (r = 0.433).

Calculation of Pearson correlation coefficients for the relationships of %FEV1, %FVC and baseline SpO_2 _with procedural nadir SpO_2 _and procedure fall in SpO_2 _demonstrated that there was a statistically significant modest correlation between baseline SpO_2 _and procedural nadir SpO_2 _(r = 0.433, p < 0.001, Figure 3), but no significant correlations found in any of the other relationships examined.

## Discussion

The main findings of this study demonstrate that flexible bronchoscopy performed in the semi-recumbent posture leads to decreased cough rate and sedation use during the initial phase of the procedure as compared with the supine posture. Based on VAS questionnaire results, the semi-recumbent posture may also result in greater patient comfort. Additionally, examination of factors which may predict a lower procedural nadir SpO_2 _or fall in SpO_2 _show that a significant correlation exists between baseline SpO_2 _and nadir SpO_2_, but that there is no correlation with either %FEV1 or %FVC.

No previous studies have examined the effect of bronchoscopy posture on coughing, patient comfort, or sedative medication use. Increased cough and discomfort when supine may be caused by a number of factors, including greater pooling of secretions in the upper airway, impaired mucociliary clearance [15], and exacerbation of gastro-oesophageal reflux [16]. Anecdotally, we previously found that patients tend to cough less in the semi-recumbent posture and hence may be more comfortable. This study quantified coughs and reported a significantly lower cough rate and sedative requirement, and semi-quantitatively indicated better patient comfort when compared with the supine posture.

There have been limited studies on differences between flexible bronchoscopy performed in different postures. These previous studies have focused on the occurrence of hypoxemia. Meghjee et al [11] randomized patients to commencing bronchoscopy in the supine or semi-recumbent posture, and altered postures during the procedure. They found that sedation induced significant oxygen desaturation but alterations in posture did not, and that oxygen supplementation at 2 L/min restored saturations to pre-sedation levels. In a more recent study, van Zwam et al [10] randomized patients to the supine or sitting postures, and unexpectedly found that the sitting posture resulted in more frequent instances of 4% oxygen desaturation. Somewhat contradictory to this, although more expectedly, another study reported that patients with lower FEV1 experienced more prominent desaturation in the supine position [12]. The reasons behind the findings of the former study are unclear, and the authors postulated that ventilation-perfusion matching may be better in the supine posture; although it has been shown that such matching generally worsens when changing from sitting to supine [17,18].

That oxygen saturation falls during flexible bronchoscopy and is correctable with supplemental oxygen is widely recognised [6,7,11,19,20]. Mechanisms that contribute to this include decreased respiratory drive and hypoventilation from sedative use [21], ventilation-perfusion mismatch [9], reduction in lung function [22], and partial airway obstruction [23]. It can be expected that bronchoscopy performed in the supine posture would predispose to greater degrees of oxygen desaturation, given that lung function will be further compromised [24,25] and that ventilation-perfusion matching may worsen [17,18]. Our findings show, however, that when bronchoscopy is performed with oxygen supplementation commenced at 2 L/min, there is no significant difference in the degree of oxygen desaturation or oxygen supplementation required between supine or semi-recumbent postures.

Patients with impaired lung function experience greater degrees of oxygen desaturation during bronchoscopy [9,12] and patients with severe COPD in particular are known to experience higher complication rates [26]. Unexpectedly, there was no significant correlation between %FEV1 or %FVC with either the procedural nadir SpO_2 _or fall in SpO_2 _in our study. There was a significant correlation between baseline SpO_2 _and procedural nadir SpO_2_, although the lack of correlation of baseline SpO_2 _with the fall in SpO_2 _is again unexpected, as patients with a lower baseline SpO2 should desaturate to a greater degree given their position on the oxyhaemoglobin dissociation curve [27].

The use a 100 mm VAS in the setting of acute cough has recently been studied, and the minimum important difference was identified as 12 mm [28], which compares closely with the use of VAS in other settings such as pain [29] and nausea [30]. This difference was observed in nurse-perceived patient discomfort and in patient-perceived cough, although the latter was not statistically significant. However, it is important to note that nursing staff and patients were not blinded to the posture adopted, and there is likely to be a degree of inter-observer variability between nursing staff, hence potential biases in the results cannot be excluded.

We limited our observations to a defined initial phase in order to achieve a standardized period with a well defined procedure protocol. This resulted in a narrow range of observation times (see Table 1) and mitigates the effects of variable numbers and types of diagnostic maneuvers that may confound outcomes, such as bronchoalveolar lavage which would induce further oxygen desaturation [31,32]. Patients were not randomized by bronchoscopy posture and procedures were conducted based on operator preference, which we acknowledge has the potential to introduce various biases, including selection, operator, observer, and recall bias. However, different operators staff each procedure list and frequently perform procedures on patients for whom they are not the treating clinician. Hence, this provided a degree of randomness to the allocation of patients to operators. Furthermore, three operators routinely perform the procedure in both postures, and elected to alternate between the supine and semi-recumbent postures during the study. Examination of results for these operators revealed the same trends as for the study as a whole (see results section), indicating that the differences observed may truly be due to the effect of posture.

In summary, the semi-recumbent posture for flexible bronchoscopy results in reduced cough and sedative medication use, and is likely to result in greater patient comfort. Whilst it is unlikely that causation of cough during bronchoscopy will have long term consequences, less cough may be advantageous when performing diagnostic procedures such as endobronchial biopsies or transbronchial needle aspiration, and the semi-recumbent posture should be considered when such procedures are necessary. Less cough also reduces the need for escalating sedative drug doses to allow better control during bronchoscopy. The lower sedative requirements may reduce the risk of significant respiratory depression particularly in patients with predisposing respiratory diseases. Additionally, greater procedural comfort will always be appreciated by patients.

## Competing interests

The authors declare that they have no competing interests.

## Authors' contributions

All authors contributed to conception, writing and critical review of the manuscript. ITL, FP & SAM performed procedures and collected data related to the study. ITL & FP performed literature searches and statistical analyses. All authors read and approved the final version of the manuscript.
